# Breaking Bad News During Prenatal Screening: The Role of Professional Obstetricians and Midwives in Greece

**DOI:** 10.7759/cureus.56787

**Published:** 2024-03-23

**Authors:** Anna Glynou, Dionysios G Galatis, Vassilis Yalelis, Alexandros Sotiriadis, Andreas Pampanos, Angeliki Sarella, Eirini Chasalevri, Maria Koukaki, Panagiotis M Peitsidis, Makarios Eleftheriades

**Affiliations:** 1 Department of Midwifery, Maternity Hospital Elena Venizelou, Athens, GRC; 2 Department of Obstetrics and Gynecology, National and Kapodistrian University of Athens School of Medicine, Athens, GRC; 3 Department of Obstetrics and Gynecology, Maternity Hospital Elena Venizelou, Athens, GRC; 4 Department of Obstetrics and Gynecology, Ippokrateio Hospital of Thessaloniki, Thessaloniki, GRC; 5 Department of Genetics, Alexandra General Hospital, Athens, GRC; 6 Department of Midwifery, Faculty of Health and Caring Sciences, University of West Attica, Athens, GRC; 7 Department of Obstetrics and Gynecology, Hygeia IVF Embryogenesis, Athens, GRC

**Keywords:** informed consent, obstetricians, midwives, prenatal screening, breaking bad news

## Abstract

Introduction: Breaking bad news is one of the most difficult responsibilities in medical practice. Although medical staff in clinical practice often encounter situations that necessitate the announcement of unpleasant news, there is a lack of training regarding their communication with patients and their families. Effective interaction between medical staff and pregnant women constitutes a crucial component of breaking down unpleasant news. This research aimed to investigate the knowledge and attitude of health professionals, particularly obstetricians, and midwives, regarding the announcement of bad news during prenatal screening.

Methods: The study was conducted between September 2017 and April 2018. One hundred professional obstetricians and midwives involved in fetal and prenatal medicine in Greece were part of the study. The study consisted of two parts: the first covered the emotional state of healthcare professionals during the announcement of unpleasant news, and the second covered the appropriate way to inform unpleasant results during prenatal testing.

Results: In this study, only 41% of the participants considered that they felt comfortable discussing issues related to the diagnosis of an unpleasant result during prenatal testing with the pregnant woman/patient, or her relatives, and 85% accepted that they had experienced feelings of sadness, anxiety, or guilt when announcing unpleasant results. Furthermore, 87% of the participants believed that the non-verbal communication component (eye contact, body language) plays an important role in breaking bad news. Finally, 65% considered that prolonged monitoring of the ultrasound screen during prenatal screening does not increase the anxiety of pregnant women when carried out for a better medical opinion.

Conclusions: Delivering bad news during prenatal screening creates stress for the parents. As far as the ethical, cultural, psychological, and legal complicity of healthcare professionals is concerned, communicating unpleasant news has been a subject of discussion by many experts. It is important to understand the concerns of women regarding the risks of counseling.

## Introduction

In the field of obstetrics, prenatal screening gives important information regarding the fetus. Ultrasound is a standard procedure performed during pregnancy and is described as a very positive experience by nearly all mothers and fathers [1]. Based on several studies, communicating unpleasant news to patients is a crucial factor in creating an exceptional bond between them and healthcare professionals [2]. Effective interaction between medical staff and pregnant women constitutes a crucial component of breaking unpleasant news. Furthermore, according to various studies, patients tend to be significantly more interested in finding out as much information as possible regarding their diagnosis, details of their condition, therapy methods, and an approximate estimate of their life expectancy [3].

Ultrasound screening is an important examination during pregnancy and is an accepted tool that undoubtedly helps in the diagnosis of many prenatal diagnostic findings. In most cases, an abnormal finding is often unexpected and usually comes as a shock to parents [4]. The expectation created by ultrasound examination is mainly explained as a positive feeling for the parents. Thus, most of the time, they are not prepared when the findings are not normal, and negative feelings are created among the couples. All parents know that the ultrasound assists healthcare professionals detect several abnormalities in the fetus during prenatal screening, although most believe that this is not the main purpose of the examination [4].

During the ultrasound examination, healthcare professionals explain their findings to the pregnant woman, giving information mainly about the fetus. When potential problems or abnormalities are found in the embryo, professionals experience a difficult situation, and despite remaining silent, they may unintentionally communicate their concerns through their body language. The attempt to convey an unpleasant event silently, without words, many times leads the patient to the conclusion that something is wrong. Reacting to bad news follows a specific path: after the shock or disappointment comes denial, followed by dissent, sadness, or depression, and at last acceptance. In the majority of cases, the shock during the first phase can create difficulties for the pregnant woman to understand the information given to her [5].

During pregnancy, women frequently experience significant emotional reactions, such as fear, guilt, sadness, and hopelessness. Additionally, it is worth noting that both parents often strongly experience isolation, anxiety, and insecurity. Specifically, while the fetal ultrasound examination is taking place, women experience grief as opposed to men, who in most cases express anger, rage, and anxiety [4].

The term ‘breaking bad news’ is mostly associated with the moment when healthcare professionals are obliged to announce negative medical information to a patient or their relatives [6].

Usually, the first reactions expressed by patients include shock, surprise, disbelief, and denial, followed by feelings of frustration, anger, loss of control, and anxiety. Subsequent reactions often appear after a while, containing feelings of guilt, embarrassment, disappointment, isolation, depression, grief, and mourning [7].

## Materials and methods

This study was conducted using a questionnaire created for the purpose of examining the attitude and knowledge of healthcare professionals, particularly obstetricians and midwives, in the obstetric process concerning the announcement of unpleasant news during prenatal screening. Afterward, a statistical analysis was performed with a quantitative method using a small number of respondents in order to investigate any unusual points occurring during the study.

The research was carried out between September 2017 and April 2018 in prenatal care centers and obstetrics clinics, trying to integrate as many people as possible to include a variety of age and educational profiles. A total of 100 health professionals (obstetricians, gynecologists, and midwives) took part in the questionnaire. Before completing the questionnaire, participants were informed both orally and through a provided written informative study sheet regarding the content and goals of the study. The survey was approved by the National Health System General and Maternity Hospital “Helena Venizelou” in Athens in September 2017 (approval number: 9/2017).

The questionnaire was completed by 100 health professionals (obstetricians, gynecologists, and midwives) and consisted of two sections. Part one covered the participants' general opinion about announcing unpleasant results during prenatal testing and trying to describe their feelings of sadness, anxiety, or guilt that they were experiencing when they informed the couples about all the implications regarding the diagnosis. Part two assessed the background of healthcare professionals' attitudes and knowledge of the announcement of bad news during prenatal care. 

The data were expressed as frequencies and percentages for categorical variables. A comparison between midwives and doctors in relation to the categorical variables was performed using Fisher's exact test. All tests were two-sided, and statistical significance was set at p <0.05. All analyses were carried out using IBM SPSS Statistics for Windows, version 21.00 (IBM Corporation, Armonk, NY).

## Results

The sample consisted of 100 healthcare professionals (50 midwives and 50 obstetricians and gynecologists). Thirty-one percent of the participants were men, and 69% were women. Seventy-five percent were between 25 and 45 years of age, and 25% were between 46 and 65 years of age. Fifty-eight percent were university graduates, and 28% were postgraduates. The demographic and clinical characteristics of the sample are given in Table 1.

**Table 1:** Demographic and clinical characteristics of the sample

Particulars		N	%
Sex	Men	31	31%
	Women	69	69%
Age (years)	25-35	40	40%
	36-45	35	35%
	46-55	16	16%
	56-65	9	9%
Educational level	University: technological education	58	58%
	Postgraduate education	28	28%
	Holder of a doctorate or post-doctoral degree	14	14%
Specialty	Obstetric staff	50	50%
	Medical staff	50	50%

Table 2 presents the results of their attitude and knowledge regarding the announcement of bad news during prenatal screening.

**Table 2:** Professionals’ attitude and knowledge on the announcement of bad news during prenatal screening All values are presented as frequencies (percentages) of positive answers.

Questions	Midwives	Doctors	p-value
Are you comfortable discussing issues regarding the diagnosis of an adverse result during prenatal testing with the patient/relative?	16 (32 %)	25 (50.0 %)	-
Have you experienced feelings of sadness, anxiety, or guilt when reporting unpleasant results?	46 (92.0 %)	39 (78.0 %)	-
Which of the following two situations do you consider most stressful for your psychology when informing the patient?			
Bad prognosis	21 (42.0 %)	27 (54.0 %)	0.317
False hope	29 (58.0 %)	23 (46.0 %)	
Do you think that in cases of very unpleasant results, the partner or relatives should be informed first in order to achieve better management of the news by the pregnant woman/patient?	19 (38.0 %)	10 (20.0 %)	-
Do you think that the patient should be informed of all the ramifications of the diagnosis of an unpleasant result during prenatal testing, at the time of diagnosis?	25 (50.0 %)	27 (54.0 %)	0.841
Initial announcement of the unpleasant news and then an update on a second visit.	19 (38.0 %)	14 (28.0 %)	0.395
Partial information at each visit, as long as the medical condition allows.	6 (12.0 %)	6 (12.0 %)	1
If the relatives, in case they have been informed first, want to hide the diagnosis, do you agree with the relatives and avoid difficult questions?	1 (2.0 %)	2 (4.0 %)	1
You inform the relatives that you do not agree with this treatment and suggest a change of approach.	37 (74.0 %)	30 (60.0 %)	0.202
You completely disagree with the relatives and inform the patient.	12 (24.0 %)	19 (38.0 %)	0.194
The appropriate way of informing in cases of unpleasant results during prenatal testing is verbal with complete and clear information.	1 (2.0 %)	3 (6.0 %)	0.617
The appropriate way of informing in cases of unpleasant results during prenatal testing is verbal with full details, addressing the patient's emotional reaction.	5 (10.0 %)	7 (14.0 %)	0.76
The appropriate way of informing in cases of unpleasant results during prenatal testing is verbal with full details, addressing the patient's emotional reaction, accompanied by discussion, and providing future planning.	45 (90.0 %)	43 (86.0 %)	0.76
The place for the announcement of any unpleasant news should be specific, for example after the examination in a bright clinic and in the presence of other medical staff with a view to maintaining confidentiality.	39 (78.0 %)	34 (68.0 %)	-
While informing the patient, the vocabulary should be fully medical to rigid, even if some terms may not be fully understood by the patient.	1 (2.0 %)	1 (2.0 %)	1
While informing the patient, the vocabulary should be medical, but with the possibility of switching to a more comprehensible vocabulary.	30 (60.0 %)	28 (56.0 %)	0.84
While informing the patient, the vocabulary should be in everyday vocabulary to achieve communicative coupling with the patient and an emotional approach.	20 (40.0 %)	21 (42.0 %)	1
The most important reason for breaking bad news to a patient is that the patient can be helped to improve coping strategies.	13 (26.0 %)	10 (20.0 %)	0.635
The most important reason for breaking bad news to a patient is that it is part of the job.	5 (10.0 %)	5 (10.0 %)	1
The most important reason for breaking bad news to a patient is that it is the patient's absolute right to know.	34 (68.0 %)	35 (70.0 %)	1
The most important reason for breaking bad news to a patient is that it is unethical not to tell patients the truth.	8 (16.0 %)	6 (12.0 %)	0.774
The most important reason, in your opinion, for not breaking bad news to a patient is the possible worsening of their health condition.	26 (52.0 %)	19 (38.0 %)	0.228
The most important reason, in your opinion, for not breaking bad news to a patient is that patient denial is paramount.	4 (8.0 %)	8 (16.0 %)	0.357
The most important reason, in your opinion, for not breaking bad news to a patient is that the patient has the right but not the obligation to hear bad news.	4 (8.0 %)	3 (6.0 %)	1
The most important reason, in your opinion, for not breaking bad news to a patient is when the patient is a minor.	18 (36.0 %)	22 (44.0 %)	0.541
Prolonged viewing of the screen during antenatal screening should be avoided as it contributes to worsening the anxiety of the pregnant woman/patient.	3 (6.0 %)	11 (22.0 %)	0.041
To improve the process of breaking bad news, the medical staff should warn the patient in advance that they have bad news so that the patient has sufficient time to process it.	3 (6.0 %)	3 (6.0 %)	-
To improve the process of breaking bad news, the medical staff should use clear, simple, and understandable vocabulary.	14 (28.0 %)	15 (30.0 %)	-
To improve the process of communicating bad news, the medical and nursing staff should check whether the communication of information is understood by the patient.	5 (10.0 %)	14 (28.0 %)	-
To improve the process of breaking bad news by the medical staff, there should be a short break to allow the patient time for any reaction or questions.	14 (28.0 %)	7 (14.0 %)	-
To improve the process of breaking bad news by the medical staff, the patient should be asked if any family members will be present.	3 (6.0 %)	2 (4.0 %)	-
To improve the process of communicating bad news by the medical and nursing staff, the doctor should be focused on the patient's problem with his/her undivided attention.	11 (22.0 %)	9 (18.0 %)	-

In our research, only 41% of the participants considered that they felt comfortable discussing with the pregnant woman/patient or her relatives issues related to the diagnosis of an unpleasant result during prenatal testing, and 85% accepted that they had experienced feelings of sadness, anxiety, or guilt when announcing the unpleasant results. Upon being asked what they considered most stressful for their psychology when informing the pregnant woman, 52% of the participants answered "false hope" in particularly unpleasant situations, and 48% replied "bad prognosis." In cases of very unpleasant results, 29% of the participants believed that the partner or relatives should be informed initially in order to achieve better management of the news by the pregnant woman.

All participants (100%) considered that the pregnant woman should be informed about all the implications regarding the diagnosis of unpleasant results during the prenatal screening. Fifty-two percent of the participants thought that this overall information should be given at the time of diagnosis, and 33% thought that the information should be given initially at the time of diagnosis and then at a second visit. Sixty-seven percent of the participants declared that if the relatives had been informed first and wanted to hide the diagnosis, the participants would disagree but would suggest a different approach to the relatives, while 31% would completely disagree with the relatives and would proceed to inform the pregnant woman.

Eighty-eight percent of the participants considered that the appropriate way of informing in cases of unpleasant results during the prenatal screening was verbal with full details, dealing with the emotional reaction of the pregnant woman/patient, accompanied by a discussion and provision of future planning. Seventy-three percent believed that the place of announcing any unpleasant news should be a specific room, for example, after the examination, in a bright clinic, with the presence of other medical staff, in order to maintain confidentiality.

Fifty-eight percent of the participants thought that the vocabulary used while informing the pregnant woman/patient should be medical, but with the possibility of switching to a more comprehensible one, and 41% thought everyday language should be used to achieve a true communicative connection with the pregnant woman based on empathy and an emotional approach. Eighty-seven percent of the participants believed that the non-verbal component of communication (eye contact, body language) also plays an important role in breaking bad news.

When asked what the most important reason was for breaking bad news to a pregnant woman, 69% believed that it was the pregnant woman's/patient's absolute right to know the specifics regarding their condition, and 23% believed that the pregnant woman/patient could be helped in terms of improving coping strategies. Forty-five percent considered that the most important reason for not announcing unpleasant news to a pregnant woman was the possible deterioration of her health condition, and 40% was in the case that the pregnant woman was a minor.

Sixty-five percent considered that prolonged viewing of the ultrasound screen during a prenatal screening does not pose a parameter for increasing anxiety in the pregnant woman if it is carried out in the context of the best medical practice (also, 21% answered negatively).

The participants considered it imperative to improve the process of announcing bad news by the medical and midwifery staff. Twenty-nine percent of the participants declared that the language used during the announcement must be clear, simple, and understandable. Twenty-one percent thought that there should be a short break after the announcement of the unpleasant news so that the pregnant woman can have the appropriate time for any reaction or for questions, and 20% thought that the health professional or doctor should be focused on the problem of the pregnant woman with his undivided attention.

Eighty-one percent considered that they had not received the required and sufficient training regarding the management of unpleasant results in the context of informing the pregnant woman. Eighty-nine percent believed that special seminar-type training for the announcement of unpleasant results during prenatal care should be addressed to the medical and nursing staff at hospitals or clinics.

## Discussion

Over the last 30 years, the value of ultrasound screening in the prenatal diagnosis of fetal abnormalities and the assessment of fetal growth and wellbeing has undoubtedly been demonstrated [8]. Nowadays, in many countries, prenatal screening tests are offered to pregnant women as part of standard prenatal care. These tests provide crucial information about the pregnancy and assist in calculating the chances of the fetus carrying a particular abnormality [1]. Most pregnant women in the first trimester have an ultrasound screening to accurately determine their gestational age [9]. At the 12th week of pregnancy, nuchal translucency (NT) measurement is offered, which is an efficient indicator for chromosomal anomalies, carrying an impressive detection rate of 80% for Down syndrome [10].

The definition of bad news is information that points towards the consideration of an unfavorable outcome [11, 12]. In obstetrics, bad news can be the precursor to adverse effects on the health of the mother or the fetus, defying hopes and expectations [13-16]. Many emotions, potentially negative ones, are experienced during the transmission of bad news, both from the pregnant women and from the medical staff [11, 13-16]. The discovery of a fetal anomaly or fetal dysplasia on ultrasound is most commonly an unexpected, particularly stressful, and sometimes emotionally devastating event for pregnant women. Healthcare professionals face a particularly difficult challenge in counseling these women about the ultrasound findings. Such a demanding task, of course, requires empathy and sensitivity [17].

The announcement of unpleasant news is mostly associated with the moment when medical staff is ordered to communicate sensitive negative medical information to a patient or their relatives. However, it can also be seen as a process of interactions that take place before, during, and after bad news is broken [6].

Communicating bad news is one of the most difficult tasks healthcare professionals face in their daily practice. Research suggests that only a small percentage of obstetricians feel competent with their skills when announcing bad news. It is important to consider the patient’s needs and desires concerning the type and quantity of information provided. This will assist in providing high-quality care and help to minimize the stress and psychological trauma experienced in a bad news situation [18].

In our study, only 41% of participants accepted that they felt comfortable announcing unpleasant news during prenatal screening. Fifty percent of obstetricians claimed to feel comfortable conveying negative medical information as opposed to 32% of midwives, which indicates that the doctors in this survey are more familiar with the announcement of unpleasant news.

Healthcare professionals must be working as a team when announcing bad news. Communication can be considered a multidisciplinary activity, requiring the active involvement of different specialties [6].

Specifically in our study, 85% of the participants accepted that they had experienced feelings of sadness, anxiety, or guilt when announcing bad news, with the largest percentage of acceptance originating from the midwifery staff at 92%. It can be concluded that the announcement of unpleasant news can also affect the overall job satisfaction of employees.

When survey participants were asked which of the two-bad prognosis or false hope-they considered most stressful for their psychology when breaking bad news to pregnant women, they accepted that they were in a particularly unpleasant situation, but vacillated in their answers with “false hope," slightly predominating with 52% (58% midwives and 46% obstetricians). It is impressive that the participants took into account the parameter of incomplete information about the patient in particularly unpleasant situations, choosing to give “false hope” even though it caused them considerable anxiety.

When healthcare professionals have to break bad news, the difficulty and the dilemma of whether to tell the truth or not becomes apparent. Another purpose of providing information is that it activates patients to make well-informed and correct choices about their healthcare and plans for their future [19].

Additionally, postnatal distress levels appear to be higher when a fetal anomaly is detected between the 25th and the 30th gestational weeks. The highest levels of psychological distress were observed in women who were >22 weeks of gestational age. It is important for medical staff, doctors, and midwives to be aware of the high psychological distress levels among these women, and to individualize their care [20].

The majority of parents express doubts, uncertainty, and anxiety after being delivered certain negative information about the ultrasound findings, regardless of the seriousness and severity of the abnormality. In most cases, the initial anxiety escalates because of the environment, the dark lighting of the examination room, and the chilling sound from the ultrasound machine [4]. In our study, 73% believed that the place for announcing any unpleasant news should be a distinct, bright room in the presence of other medical staff.

The doctor is usually the giver of bad news, and the gatekeeper of information, and they take on this role because they have responsibility for any medical treatments and decisions concerning the patient [6]. In some cases, parents sense that there is something wrong with the ultrasound findings even before the midwife breaks the bad news to them. This is mainly caused by the midwife’s body language and the fact that she remained silent during the examination. The parents also eventually realize that the midwife is not permitted to discuss any specific details regarding the findings, to take the situation into her own hands, or to suggest a possible treatment [4].

All participants agreed that pregnant women should be informed about all extensions of unpleasant results during prenatal screening. Fifty-two percent accepted that the information should be announced at the time of diagnosis, with a percentage of 50% midwives and 54% obstetricians. It is reasonable that 67% of the participants considered that providing initial information to the partner or relatives is not the best management of the unpleasant news for the pregnant woman, while 31% would disregard the relatives even if they chose to conceal the information from the patient. Of course, it is not negligible, and it is possible they were affected by the strong ties that characterize the Greek family. The majority of participants agreed that they would inform relatives who disagreed with this approach and suggest a different one with a more diplomatic approach, despite the fact that the patient's self-determination, the right to be informed about one's medical condition, and confidentiality, which should contribute to the majority of the third option, were represented by 31% of the participants.

Pregnant women have been shown to experience and express much more anxiety than their spouses. This distinction in anxiety and stress between the women and their respective partners could be justified by the obvious reality that pregnant women are more likely to experience additional distress due to the pain and complications that may occur during that procedure. Another major anxiety factor is the undeniable truth that mainly the pregnant woman is responsible for carrying the fetus and undergoes all necessary invasive procedures on her own body and not her partner’s [21].

All prenatal genetic and maternal centers should offer information and guidance to pregnant women regarding the potential risk factors associated with negative pregnancy outcomes [1]. Pregnant women pay attention to the words, body language, and degree of concentration on the ultrasound monitor by the sonographer, so they may sense when something is wrong on an ultrasound even before anything is said [17]. In our study, only 14% of the participants (6% midwives and 22% obstetricians) accepted that prolonged viewing of the ultrasound screen during the prenatal examination should be avoided because it contributes to worsening the anxiety of the pregnant woman.

Some women report feeling reassured by the ultrasound and are highly appreciative of the experience. Conversely, most pregnant women have anxiety about problems the ultrasound may reveal. So, they may have some anxiety before the ultrasound screening procedure, but this anxiety decreases after the examination when no fetal abnormalities or other important problems are diagnosed [17].

Most pregnant women are not expecting bad news at the time of the obstetric ultrasound. Many women and their partners look forward to their ultrasound as a positive experience as well as an opportunity to see and bond with their baby. Pregnant women should be counseled about the indications, benefits, risks, and limitations of obstetric ultrasound [17].

The delivery of bad news is a difficult task for most medical practitioners who are not trained under a structured curriculum for that purpose [22, 23]. They are trusted to develop this ability during everyday clinical practice. Despite that, data suggest that this inexperience serves as a source of great stress that harms the channel of communication between the doctor and the patient [23, 24]. Burnout syndrome is a common result among medical practitioners [25].

Junior doctors are more likely to feel ineffective and powerless due to inadequate training [26]. The culture of “learning on the job” inherits many faulty patterns, hampering the message that should be given to the patient [26, 27]. Even though doctors frequently come face-to-face with these situations, they elect to avoid them, not being able to handle the psychological weight [28].

While the announcement of unpleasant news is known to be a source of great stress for healthcare professionals, they may underestimate the pregnant woman and her desire for information regarding the diagnosis. Most importantly, any abnormal test results should never be given over the telephone. When the tests are properly justified, an appointment must be set with medical staff to discuss the results with the pregnant woman, advising her to be accompanied by her husband or another family member or friend. Furthermore, the news should be given with the woman dressed and sitting. The doctor should sit in front of the pregnant woman, at the same height, look her in the eye, and keep in mind that most human communication is non-verbal. Body tension, hand movements, nods of the head, and frowns are perceived and interpreted by the woman [5].

In our study, both midwives and obstetricians, at a percentage of 88%, seemed to agree that the best way to deliver bad news during prenatal screening is verbally and in full detail, addressing the patient’s emotional reaction, accompanied by discussion, and providing future planning.

Verbal communication is an essential part of communicating bad news from the medical and midwifery staff, and 58% of the participants (60% midwives and 56% obstetricians) support that the vocabulary should be medical but with the possibility of switching to a more comprehensible vocabulary. Forty-one percent of the participants (40% midwives and 42% obstetricians) accepted that delivering bad news must be supported with everyday language to achieve communicative coupling with the pregnant woman.

Only 19% of the participants considered that they had received the required and sufficient training concerning the management of unpleasant results, and 89% accepted that such seminar-type training regarding the communication of unpleasant results during prenatal screening should be offered to the medical staff of the hospital or clinic where they work.

## Conclusions

In prenatal care, any healthcare professional who monitors a pregnancy will have to deliver some unpleasant news at some point. Therefore, it becomes necessary for the medical and midwifery staff to be able to deal with such situations, although communication practice does not receive special attention during medical education. Literature shows that healthcare professionals are generally not ready to share this information, and patients often have bad memories of the disclosure process, not only because of the content of the information but also because of the health professional's lack of competence. Delivering bad news during prenatal screening creates stress for parents. As far as the ethical, cultural, psychological, and legal complicity of healthcare professionals is concerned, communicating unpleasant news has been a subject of discussion by many experts. It is important to understand the concerns of women regarding the risks of counseling.
